# New Developments in the Primo Vascular System: Imaging and Functions with regard to Acupuncture

**DOI:** 10.1155/2015/142705

**Published:** 2015-08-25

**Authors:** Richard C. Niemtzow, Kwang-Sup Soh, Kyung A. Kang, John H. Barker, He Sheng Luo, Moriya Ohkuma

**Affiliations:** ^1^United States Air Force Acupuncture Center, Malcolm Grow Medical Clinics and Surgery Center, 1050 W. Perimeter Road, Joint Base Andrews, Prince George's County, MD 20762, USA; ^2^Nano Primo Research Center, Advanced Institute of Convergence Technology, Seoul National University, Suwon 443-270, Republic of Korea; ^3^Department of Chemical Engineering, University of Louisville, Louisville, KY 40292, USA; ^4^Frankfurt Initiative for Regenerative Medicine (FIRM), Experimental Orthopedics and Trauma Surgery, JW Goethe University Hospital, 60528 Frankfurt am Main, Germany; ^5^Department of Gastroenterology and Hepatology, Renmin Hospital of Wuhan University and Wuhan University Institute of Digestive and Liver Diseases, Wuhan 430-060, China; ^6^Department of Dermatology, Sakai Hospital, Kinki University, Sakai, Osaka 590-0132, Japan

The Primo Vascular System (PVS) is an enigma. Does it exist or not? But Richard C. Niemtzow has seen it at the Nano Primo Research Center, Advanced Institute of Convergence Technology under the Direction of Dr. Kwang-Sup Soh in Seoul, South Korea.

Superficial primo-vessels, also known as Bonghan ducts and Bonghan channels, were first reported in 1962 by the North Korean scientist, Kim [1]. His work is shrouded in a cloak of mystery. Scientists are not able to reproduce all of his data, or at least for the moment without substantial difficulties.

The PVS appears to be distributed throughout the entire body. Some scientists may say it is an illusion; others say it is a “new” anatomical system, while still others are convinced that these infinitesimal channels may act like optical fiber cables and transmit DNA related information continuously throughout the body using biophotons. As a comparison, we cannot see the millions of transistors on a CPU, but they are part of a manmade system that we use every day. Similarly many biological systems are indeed very minuscule. We need electronic microscopes to visualize some of their structures; otherwise, we would not know they exist.

This special issue, focused on the Primo Vascular System, contains several unique articles that challenge its understanding: in the article “Primo Vascular System: An Endothelial-to-Mesenchymal Potential Transitional Tissue Involved in Gastric Cancer Metastasis,” P. An and colleagues investigate the exciting area of gastric carcinoma and the possible role of PVS vessels as precursors of blood vessels. As such they claim that PVS may facilitate the metastatic spread of gastric cancer in the endothelial to mesenchyme tissues. The association of the PVS with cancer is one of the important discoveries by modern PVS researchers that Kim did not investigate [2].

The article “Identification of Primo-Vascular System in Abdominal Subcutaneous Tissue Layer of Rats” by C. J. Lim and colleagues describes a methodology employing Hemacolor staining to identify the PVS in rat abdominal subcutaneous tissues. The authors substantiate it by providing images. Staining methods have been critical for revealing the hard-to-observe PVS. The first effective dye used to visualize the PVS was Trypan blue [3] followed by Hemacolor [4]. Additionally, the authors present a newly developed method using Hemacolor staining to identify the PVS in the subcutaneous tissue. They explain that the importance of this observation is that these skin-PVS could be the Conception Vessels in the skin of the rats. And this may provide substantial evidence that the PVS is located at acupuncture meridians. This article provides the incentive for further research into the relationship between the PVS and anatomy [5].

The article “Fascia and Primo Vascular System” by C. Yang and colleagues reviews fascia research and its relationship with acupuncture points, meridians, and PVS. The fascia bands together the tissues in the body and is receiving attention as the basis for clinical effectors. This article provides a timely review on the relationship between basic anatomy and clinical strategies.

The article “Primo-Vascular System as Presented by Bong Han Kim” by V. Vodyanoy and colleagues discusses staining methods used by Kim [1] in his original work, which with the aid of a high-resolution microscope have subsequently been confirmed. This article provides valuable information on effective histological methods for investigating the PVS.

The article “Comparison of Alcian Blue, Trypan Blue, and Toluidine Blue for Visualization of the Primo Vascular System Floating in Lymph Ducts” by R. Cha and colleagues describes the different characteristics of these dyes that may elucidate the mechanism of preferential PVS staining.

Finally, we are evoked by the words of Konrad Lorenz, “Truth in science can be defined as the working hypothesis best suited to open the way to the next better one.”
